# Information Security Threats and Working from Home Culture: Taxonomy, Risk Assessment and Solutions

**DOI:** 10.3390/s23084018

**Published:** 2023-04-15

**Authors:** Jaidip Kotak, Edan Habler, Oleg Brodt, Asaf Shabtai, Yuval Elovici

**Affiliations:** Department of Software and Information Systems Engineering, Ben-Gurion University of the Negev, Be’er Sheva 8410501, Israel

**Keywords:** cyber security, work from home (WFH), risk assessment, threats, taxonomy

## Abstract

During the COVID-19 pandemic, most organizations were forced to implement a work-from-home policy, and in many cases, employees have not been expected to return to the office on a full-time basis. This sudden shift in the work culture was accompanied by an increase in the number of information security-related threats which organizations were unprepared for. The ability to effectively address these threats relies on a comprehensive threat analysis and risk assessment and the creation of relevant asset and threat taxonomies for the new *work-from-home* culture. In response to this need, we built the required taxonomies and performed a thorough analysis of the threats associated with this new work culture. In this paper, we present our taxonomies and the results of our analysis. We also examine the impact of each threat, indicate when it is expected to occur, describe the various prevention methods available commercially or proposed in academic research, and present specific use cases.

## 1. Introduction

During the COVID-19 worldwide pandemic, governments took steps to limit the spread of the virus and prevent health systems from collapsing. One major step taken early in the pandemic was to shut down both the private and public sectors, preventing employees from going to their workplaces and forcing them to remain isolated at home for long periods of time. This decision had major economic impact, since companies were unable to function properly, although their contracts, obligations, and expenses remained in effect.

Many companies began to explore the various options available to them, with the aim of finding a solution that would ensure their continued functioning, despite the fact that employees were unable to work on-site. The “work from home” model, which was possible but not widely used prior to the COVID-19 pandemic, was found to be the most popular solution. The work-from-home approach enables employees working from home to access the company’s network and resources using the means available to them or tools provided by the company.

Working from home places many constraints on companies, including diverting extensive resources to reorganizing company networks, adapting work practices to maintain employee effectiveness, creating mechanisms to track the delegation and execution of tasks, and of course, adopting a platform that facilitates interpersonal contact between employees and helps mitigate the difficulties that might arise due to the physical distance.

The work-from-home approach might require drastic changes to employees’ way of work. Employees were often forced to adopt new technologies, such as online meeting platforms, remote connection tools, and virtual remote machines. In this approach, employees are scattered across multiple unsupervised environments, and their communication with the company’s corporate network is performed remotely from endpoints that can easily be infected. Employees can also be targeted by phishing emails that contain malicious links for registering for online meetings; this is just one example of the many new threats introduced in the work-from-home culture. Working from home is also accompanied by many new information security threats that companies have not dealt with before. For example, the surrounding home environment introduces new challenges and threats, as home digital devices and corporate laptops are on the same network; the physical security of important devices and files is also an issue. Companies face a major challenge in encouraging employees to adopt the new technologies, along with the best practices associated with them, many of which are aimed at ensuring information security.

Despite the difficulties and risks, according to a Gartner survey [1] conducted during the pandemic, many companies plan to permit their employees to work from home partially (82%) or on a full-time (47%) basis when the pandemic subsides, since many employees see this as an advantage.

It is therefore essential to perform a risk assessment for the work-from-home culture; the risk assessment will identify the key areas of concern that need to be addressed by companies to safely adopt the new work culture. There is also a need to build asset and threat taxonomies related to the work-from-home culture to effectively map threats with assets.

In this work, we address the lack of a risk assessment and the relevant taxonomies and build two taxonomies: (i) an asset taxonomy and (ii) a threat taxonomy for the work-from-home culture. In addition to constructing these taxonomies, we provide a comprehensive analysis of the information security threats and challenges faced by companies adopting the new work culture. We also assess the degree of risk and the potential that the risk will be realized, and review the range of technologies and solutions available commercially and proposed in academic research, highlighting both the advantages and disadvantages of different solutions.

## 2. Asset Taxonomy

In order to list all of the threats and create a threat taxonomy related to the work-from-home environment, we first defined the assets in this environment, differentiating between the existing systems and networks in the home and those that the employee’s company might provide to enable remote work. The resulting list of potential assets in the work-from-home environment was used to create an asset taxonomy, which can be updated over time as new assets are added and others become obsolete. We divided the various assets into the following domains:

- ***Employees:*** An employee’s personal accessories and devices which are used on a daily basis and partially form the employee’s remote workplace. These include wearable computing devices and devices used by the employee for personal needs. Usually, these are part of a domestic network that does not include the protective measures employed in the corporate network.
- ***Wi-Fi Segment:*** Components used to connect to the network from the employee’s home, such as the Wi-Fi network segment, which is usually not secured.
- ***External Devices:*** External components that are in the employee’s home but are not part of the work environment, such as Internet of things (IoT) devices located around the house and other devices that are part of the home environment and can be connected to the employee’s computer for various purposes (for example, data transferring via computer ports).
- ***Company’s LAN Segment and Hardened Devices:*** Components used to connect to the corporate network (company’s local area network (LAN) segment) in a secure manner from the employee’s home, along with hardened/secure accessories provided by the company to enable the employee to establish an authorized connection to the corporate network.

**Figure 1:**
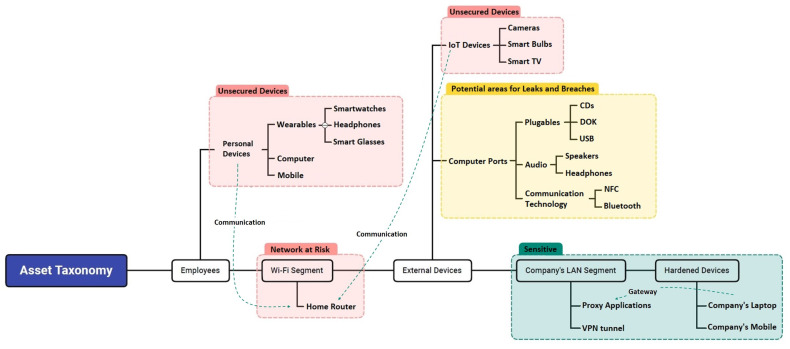
Asset taxonomy.

The assets in each of these domains can be seen in Figure 1.

## 3. Threat Taxonomy

**Figure 2:**
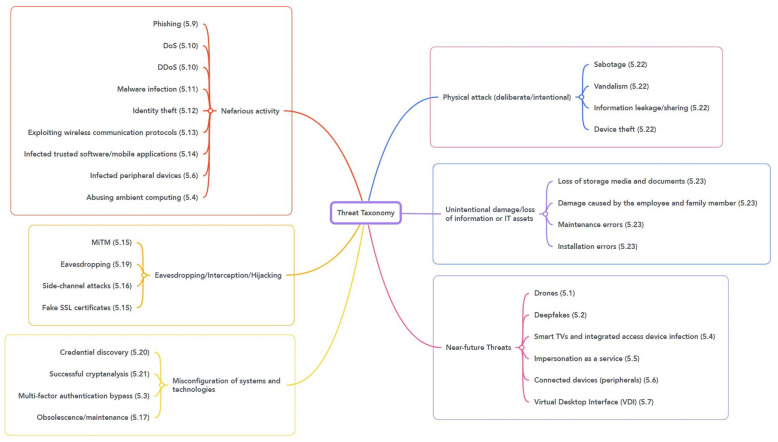
Threat taxonomy.

As a result of the work-from-home approach, many companies have changed their network infrastructures, IT, and security measures to enable connections from home and support remote users and employees. In this section, we we discuss the potential threats in a work-from-home environment. The taxonomy in Figure 2 presents the threat categories. We used the European Network and Information Security Agency’s (ENISA) threat (taxonomy https://www.enisa.europa.eu/topics/cyber-threats/threats-and-trends/enisa-threat-landscape/threat-taxonomy/view, accessed on 21 June 2022) as a starting point and modified it for the work-from-home culture.

### 3.1. Nefarious Activity/Abuse

Nefarious activities are a collection of attacks intentionally performed in order to steal information or influence an employee’s activity:

- ***Phishing***: Phishing is a type of social engineering attack often used to steal user data, including login credentials and credit card numbers. It occurs when an adversary, masquerading as a trusted entity, tricks a victim into opening an email, instant message, or text message. With the remote work scenarios common during COVID-19, phishing attacks have become one of the most significant threats faced by Internet users, organizations, and service providers. The authors of “scam pandemic” [2] describe how attackers exploit public fear through phishing.
- ***DoS***: Denial of service (DoS) is an attack affecting legitimate users by making information systems, resources, and devices inaccessible. A DoS attack is accomplished by flooding a service network/website with requests until the target cannot handle the load and the service is paralyzed. There are two common types of DoS attacks: a smurf attack and a syn attack. In a smurf attack, the adversary floods the target service by broadcasting a massive amount of packets to a number of different hosts with a spoofed Internet Protocol address that belongs to the target service/computer, thereby flooding the target with the hosts’ responses. In a syn attack, an adversary sends a massive amount of requests, preventing the service from handling the requests of other users [3].
- ***DDoS:*** Just like a DoS attack, a distributed denial of service (DDoS) [4] attack occurs when multiple entities are operating at the same time to attack a specific target. A DDoS attack can occur unintentionally when there is a massive increase in traffic in a short period of time. An example of such an effect is known as the “slashdot effect” [5], when a popular website links to a smaller website and directs many requests tow it.
- ***Malware infection***: Computers within the corporate network are usually more hermetically secured and better protected than home computers, since they are surrounded by security components (e.g., firewalls), software for monitoring and blocking malicious content, and prevention software (e.g., anti-virus software installed on the client). Moreover, internal computers enforced by domain policies and constraints, ensure software updates, enforcement of password complexity, and more. In contrast, home computers are unsupervised, and therefore, the potential of malicious infection is significantly higher. When connected to the corporate network, an infected computer can pose a threat by spreading and causing damage across the network. Common examples of malware are viruses, Trojan horses, spyware, ransomware, and exploit kits.
- ***Identity theft***: In impersonation attacks, an adversary successfully assumes the identity of one of the legitimate parties in the system and abuses the authority of the victim. By impersonating or stealing users’ identities, adversaries can manipulate both the company’s employees and its network services. There are several ways of obtaining users’ information. A phishing attack is one of the common social engineering methods. In this method, the adversary tempts the user into clicking on a malicious link, through which the adversary can damage the employee’s computer or simply steal their login credentials by impersonating a legitimate service. There are also advanced technologies, such as machine learning (ML), through which an adversary can implement a wide range of complex attacks by impersonating legitimate employees. For example, the adversary can generate a synthetic voice mimicking the victim’s manager using an ML model and trick the victim into sharing certain confidential information [6]. Recently, in order to deceive authentication systems (when risk-based authentication is applied [7]), adversaries have taken measures to resemble their targets (in terms of their web fingerprints) by connecting from the same IP address range, geographic location, and browser indicators as the target. By copying these identifiers, an adversary can connect to the corporate network without triggering an abnormal activity alert. A number of services can be found on the darknet that provide access to bundles that include these types of identifiers [8].
- ***Exploiting wireless communication protocols**:* The majority of modern smart homes utilize wireless communication protocols and as a result are vulnerable to the security threats inherent in them. The most common technology is Wi-Fi, a family of wireless protocols based on the IEEE 802.11 [9] standards, which is in use for smart bulbs, smart plugs, and more. The widespread use of these protocols makes the home router a major point of failure in the home. Moreover, Wi-Fi is vulnerable to several types of attacks, i.e., password cracking (KRACK [10]) and de-authentication attacks (WiFiphisher [11]) that can be used by disconnecting user devices from the Wi-Fi access point and then executing a man-in-the-middle attack in order to collect Wi-Fi passwords. In addition to Wi-Fi technology, Bluetooth and near-field communication (NFC) are becoming increasingly common in smart home environments, especially in low-energy and physical security systems. There are a number of known vulnerabilities in these technologies which can be used to exploit endpoint devices (e.g., the Blueborne exploit leverages Bluetooth connections to penetrate and take complete control of targeted devices [12] and remote eavesdropping attacks using an NFC receiver [13]).
- ***Infected trusted software/mobile applications**:* An adversary can reverse engineer an existing application of the company and publish a version containing malicious code, using a compromised GitHub account. This attack vector can allow the adversary to run code with high privileges and access sensitive information. An example of the use of this vector can be found in the SolarWinds supply chain attack [14], where the attackers gained access to the source code and were able to insert malicious code snippets that gave them remote code permissions.
- ***Infected peripheral devices**:* Keyboards, mouse plug-ins, and monitors can all be used for PC infection and data exfiltration. Spreading malware/a malicious payload through a signed driver represents a significant threat to peripherals. This activity requires the adversary to access the hardware manufacturer’s source code [15]. Firmware modification is another sophisticated attack vector, which relies on physical access to a target device. By interfering with a device’s firmware, it is possible to cause an infected component to infect computers connected to them. Researchers have demonstrated how a mouse can be used to infect PCs by adding malware and a utility that transfers the malware from the mouse to the target machine [16].
- ***Abusing ambient computing**:* Ambient computing is a broad term that describes an environment of smart devices, decisions, and human activity that enables computer actions alongside everyday life, without the need for direct human commands or intervention. With the use of ambient computing, companies are able to integrate technology seamlessly into many aspects of life in order to reduce the need for human attention and involvement. An example of smart home devices that try to apply ambient computing concepts are voice control systems, such as Google Assistant and Amazon’s Alexa. These high-tech remotely controlled products and technologies can be vulnerable to unwanted manipulation or activation. There have been a number of examples where third-party software (i.e., YouTube services/a TV advertisement) has been used to trigger speaker-equipped household devices to play malicious audio or perform unwanted actions, such as making purchases [17,18].

### 3.2. Eavesdropping/Interception/Hijacking

Several threats include passive/active intervention in Internet traffic. These threats require different amounts of knowledge and resources from the attacker and can provide both complete or partial information about the traffic.

- ***MiTM***: A man-in-the-middle (MiTM) attack is a general term for an attack in which an adversary secretly relays/alters the communications between two parties who believe that they are communicating directly with each other. There are several ways to perform an MiTM attack: ARP poisoning, DNS spoofing, IP spoofing, session hijacking, and Wi-Fi eavesdropping [19,20,21,22,23,24].
- ***Eavesdropping***: In an eavesdropping attack, an adversary tries to intercept, modify, or delete the data transmitted between the devices. This type of attack leverages the insecure nature of network communications to access data in transit between devices. By sniffing, an adversary can obtain information regarding the victim’s actions and their device statistics (in real time) and apply various techniques to use stolen information (e.g., phishing attacks, detection of the operating system (OS), and installed applications) [25,26].
- ***Side-channel attacks**:* Side-channel attacks allow an adversary to infer information which is not visible, by observing non-functional and physical characteristics of a program, such as computing power, communication patterns, or resource consumption. In work-from-home environments, there are many IoT devices that are prone to side-channel attacks; e.g., home routers can be easily exploited and enable the adversary to analyze and identify traffic and infer sensitive information (even if the communication is encrypted [27,28]). Even virtual-private-network (VPN)-tunneled traffic is prone to similar attacks [29]. Advanced side-channel attacks, such as the Lamphone [30] and Glowworm attacks [31], use physical properties such as changes in a light bulb’s frequency in response to sound and optical emanations from a device’s power indicator LED to eavesdrop sound.
- ***Fake SSL certificates***: SSL certificates are provided by a trusted certificate authority (CA). When someone applies for an SSL certificate for their business, the CA verifies the information provided. Once verified, the CA provides the SSL certificate with a signature. An adversary can generate a fake SSL certificate by self-signing and installing the root certificate on the victim’s machine (using tools such as BurpSuite) or obtaining the username and password of a legitimate CA and signing its certificate request. By using fake SSL certificates, an adversary can read transferred information and perform phishing attacks (illustrated in [32,33]).

### 3.3. Misconfiguration of Systems and Technologies

The adoption of remote work requires reconfiguration of a company’s network and security components, in addition to the adoption of new technologies. Therefore, errors and inaccuracies in configurations and installations could occur. Moreover, employees can also inadvertently perform configuration errors on their personal computers. As a result of weak configurations, many errors could occur:

- ***Credential discovery**:* Disclosure of information regarding credentials may occur due to weak security measures, implementation of the applications, or settings chosen by the user—e.g., in the Mozilla Firefox web browser, there is an option to set up a primary password to protect stored logins and passwords, and in the default settings, passwords are saved as cleartext [34].
- ***Successful cryptanalysis**:* Cryptanalysis [35] is a process of finding weaknesses in cryptographic algorithms and using these weaknesses to decipher the ciphertext without knowing the secret key. Sometimes, the weakness is not in the cryptographic algorithm itself but rather in how it is applied by the application. An adversary may have other goals as well, such as determining the secret key, finding a functionally equivalent algorithm for encryption and decryption that does not require knowledge of the secret key, or gaining information about plaintext or ciphertext that was not previously known; alternatively, they may want to distinguish the output of the encryption (ciphertext) from a random permutation of bits.
- ***Multi-factor authentication bypass**:* Multi-factor authentication (MFA) adds a layer of protection to the sign-in process. When accessing accounts or applications, users provide additional identity verification, such as scanning a fingerprint or entering a code sent to his or her phone. However, there are certain techniques among adversaries that are used to bypass MFA. Popular techniques [36] include: **(i) Manipulating architectural and design flaws:** In this technique, an adversary can leverage the compromised credentials of user A to access the VPN tunnel and then later try to log in to different services using user B’s credentials. **(ii) Exploiting insecure token on-boarding processes:** An adversary can obtain the URL from the victim’s email, which is used to pair the MFA token application of the phone with authentication server (this is usually shared when an employee joins an organization). The same link, if not expired, can be used to pair the adversary’s phone with authentication server to generate a new one-time password (OTP). **(iii) Attacking browser cookies post-authentication:** An adversary can obtain cookies from the compromised browser of a victim and reuse them later on a different computer to access the services whose cookies were obtained. **(iv) Targeting critical assets through secondary channels:** In active directory (AD) environments, remote management ports are enabled by default; other protocols, such as Server Message Block (SMB) and Remote Procedure Call (RPC), can be accessed with tools such as PsExec, Powershell, and other direct component object model (COM) objects. These protocols are exempt from two-factor authentication (2FA), as most MFA modules do not cover non-interactive communication. In this case, the adversary would be able to log in and gain access to the server using only a username and password. **(v) SIM swapping/hijacking:** Here, an adversary takes over the mobile phone number of the real subscriber, i.e., victim, by asking the mobile telecom provider to link that number to a SIM card under the adversary’s control. When the attack is successful, the victim’s phone will lose its connection to the network, and the victim will be unable to make or receive phone calls. The adversary takes over the account and can receive all SMS and voice calls intended for the legitimate subscriber.
- ***Obsolescence/maintenance**:* Obsolescence of software and equipment beyond its supported service life might occur unintentionally, when a user fails to pay attention to required updates or upgrades. Obsolescence may result in a lack of maintenance and lead to components that can no longer perform their function due to incompatibility; such components will also be exposed to known attacks that have been made over the years.

### 3.4. Physical Attack (Deliberate/Intentional)

In the work-from-home environment, due to the decentralization of work, the employee’s physical security is of concern. Unlike the traditional workplace where crucial digital assets are in a highly secured centralized environment, in the work-from-home environment, digital devices and sensitive documents are not in a highly secured setting, and therefore, are at increased risk of being the targets of a physical attack. The following threats are associated with a physical attack:

- ***Sabotage**:* Sabotage is a set of actions performed by an adversary to intentionally interfere with the victim’s ability to perform tasks (non-fulfillment or defective fulfillment). It can include damage to digital assets, such as the computer or home router, with the aim of making it difficult for the victim employee to perform a routine task. The likelihood of attack increases when working from home, since the victim’s home is less secure than the corporate office. In this way, the decentralization of the workforce adds risk.
- ***Vandalism**:* Vandalism is very similar to sabotage, where an adversary’s intention is to harm the victim’s digital assets. In sabotage, an adversary has a particular motive for causing damage to digital assets, whereas in vandalism the motive is limited to harming the digital assets.
- ***Information leakage/sharing**:* As the home of the employee is now the office, it may contain sensitive information in the form of physical documents or on thumb drives to which an adversary may have easier access compared to the corporate office; this can lead to the leakage of sensitive information.
- ***Device theft**:* There is also a possibility that a thief can steal valuable digital assets, such as a laptop from the employee’s home for monetary gain. The likelihood of this threat is greater in a work-from-home setting than in a corporate environment.

### 3.5. Unintentional Damage/Loss of Information or IT Assets

The use of IT devices in the home environment may leave them vulnerable to external events and adversaries. IT devices can suffer from information fossilization or destruction of components that will prevent the employee from working. The following are the main threats in this area:

- ***Loss of storage media and documents***: When the work desk shifts from the corporate office to the employee’s home, the surrounding environment also changes. In a work-from-home setup, there are additional people, such as family members and visitors. They too gain access to the things that are placed on or near the home work desk. There is also a chance that some documents, storage drives, etc. could be misplaced by other people in the home. Even children, while playing, can misplace important items that are related to work.
- ***Damage caused by the employee of family members***: The work-from-home culture makes employees more comfortable, and there may be more casual behavior at home, even during office hours. While working, employees might eat or drink at the same table where their laptop is placed and could spill or drop food on the laptop/documents, which might cause damage to such items.
- ***Maintenance errors***: Corporate computers receive regular updates to keep the devices up to date. Often, due to an unstable Internet connection at home during online installation, instability in connection can cause an error that might be time consuming to address or can put the computer in an unwanted state. The chances of these kinds of errors occurring are higher at home than in the corporate office.
- ***Installation errors***: Often, due to an employee’s lack of awareness of installation policies or limited admin privileges, an employee can attempt to install some required applications but fail to do so properly. In the corporate environment, an employee can quickly reach out to the help desk or support staff in person to resolve the issue. However, it can be difficult to explain the issue to remote support staff in the work-from-home setting, and it might take longer than usual. In addition, an error in installing unknown/untrusted software might temporarily prevent a user from performing certain activities due to suspicion.

### 3.6. Near-Future Threats

In this section, we present some threats that will be more prominent in the near future:

- ***Drones***: Today, attacks using drones are a matter of concern due to the lack of government standards for drone use. For an adversary, drones represent an inexpensive means of getting closer to a victim while remaining anonymous and without being caught physically. There are many ways in which drones can be used by an adversary to accomplish malicious tasks. For example, a drone can be used to drop a mini computer (such as a Raspberry Pi) near the victim’s home, which could be used to hack or monitor the victim’s Wi-Fi [37,38]. A drone can also be used to hijack Bluetooth peripherals, such as mice and keyboards and IoT devices connected to Wi-Fi [39]. Keylogging would enable a drone-mounted computer to steal passwords from users. Similarly, drones equipped with a software-defined radio that are located near a TV antenna can transmit a signal that is more powerful than the one broadcasted by legitimate TV networks, overriding the legitimate signal and displaying adversary-owned video on smart TVs in order to perform phishing attacks [40]. Drones can fly in the proximity of the victim’s computer and mimic a fake wireless printer and obtain a file that a victim intended to print [41]. A drone can also be used to drop malicious thumb drives in the home of a victim, which, when inserted into the victim’s personal computer/laptop could infect it. Drones can also be used to monitor the screen of a victim’s TV/laptop; an adversary can also record the victim at sensitive times in order to blackmail them [42].
- ***Deepfakes***: The term deepfake is typically used to refer to a multimedia item that has been edited using an algorithm in order to replace the attributes of the person in the original multimedia file with those of someone else in a way that makes the multimedia piece look authentic. Deepfakes are mainly applied to generate synthetic video, audio, and text. An adversary can leverage publicly available tools such as DeepFaceLive [43], Avatarify [44], Faceswap (https://faceswap.dev/, accessed on 21 June 2022), and others to generate a synthetic video or real-time stream of a key person within an organization and publish it on social media platforms. There are tools such as real-time voice cloning which are capable of generating any synthetic voice given a transcript with a sample as small as five seconds [45]. Tools such as voice.ai https://voice.ai/ (accessed on 21 June 2022) provide live voice-to-voice dubbing using AI. Combining such synthetic audio and video of a key person performing unintentional actions can create a more impactful video that elicits viewer trust. Similarly, AI can be used to learn the text formatting of a victim and generate synthetic text for misuse without the victim’s awareness. Various deepfake use cases are listed in Table A1.
- ***Smart TVs and integrated access device infection***: Smart TVs are devices with great potential for cyber vulnerabilities that can be exploited to compromise a person’s privacy, since they contain built-in cameras and microphones. These devices, which are connected to the Internet directly or through an integrated access device, can be infected by a wide range of attack vectors and used for spying and leaking information. There are number of ways to infect a smart TV, locally and remotely:
  1. ***Local attacks:*** The operating systems of smart TVs are not very different from those of computers and are therefore, exposed to the same risks. There is already a wide range of known vulnerabilities; openLGTV https://openlgtv.github.io/ (accessed on 21 June 2022) is an example of a reverse engineering project used to find vulnerabilities. In addition, the integrated access device (IAD) connected to the TV is also vulnerable to a variety of attacks [46] and can be used as an access point to the TV and home network.
  2. ***Remote interface range attacks:*** In smart TVs, the broadcast interface is always on, and there is no way of turning it off. Moreover, there is no authentication of any kind, and the data coming from the radio interface are considered trusted by receivers. Therefore, an adversary can abuse lack of authentication mechanism to display any video. The relevant interfaces are the asymmetric digital subscriber line (ADSL), which is a technology that provides high transmission speeds for video and voice to homes over an ordinary copper telephone wire and the digital video broadcasting (DVB) range, which is a set of international open standards for digital television. Researchers have demonstrated such remote attacks on smart TVs in the past [47].
- ***Impersonation as a service***: In order to authenticate users remotely, risk-based authentication is widely adopted as a means of evaluating whether the authenticating user has already connected to the service with the same identifiers and trusted connection (e.g., same device, IP address, location, and browser). During the authentication process, the risk-based authentication technique monitors suspicious login attempts and raises an alert triggering the multi-factor authentication (MFA) process in the case of suspicious login attempts. Recently, in order to bypass the MFA utilities, adversaries began trying to impersonate legitimate customers/employees in order to connect to the company network by using behavioral identifiers that characterize those customers/employees. A popular service described by [8] showed that a paid service can be used to acquire an accurate set of profiles of legitimate employees in a large number of companies. These profiles have been collected by malware. The service provides bundles including all of the relevant identifiers of the employees, thereby enabling an adversary to bypass the risk-based authentication model.
- ***Connected devices (peripherals)***: Peripherals include both internal and external devices. Internal peripherals are built into a computer by the manufacturer (e.g., video and sound cards, internal modems, and hard disk drives). External peripherals are connected either by cables, such as a universal serial bus (USB) cable, or directly to the host device’s port, or even wirelessly using Wi-Fi or Bluetooth. There are a variety of options available to an adversary interested in exploiting these components to intrude on a computer system.
  1. ***Drivers**:* The OS uses programs called device drivers to manage connections with peripherals. By altering the driver’s code, which is installed when the device is connected to the computer, various malicious operations can be performed using the high permissions of these drivers. Since all drivers running on Windows must be signed before Windows will load them, the adversary has to infiltrate in the supply chain and compromise this software while it is still at the manufacturer. In the past, gaming mouse-maker, Razer, was the victim of such an attack [15].
  2. ***Firmware**:* Threat actors can use the device firmware (the software that controls the device hardware) to run rootkits, a type of software that masks itself and hides malware on a device. This type of software enables threat actors to remotely control devices. “Mousetrap” [16] is an example of a firmware rootkit used to infect PCs.
  3. ***Cable manipulation**:* Smart connection cables (e.g., lightning and USB-C) have small microcontrollers embedded in them. Adversaries can program these microcontrollers, enabling them to attack a device when it is plugged in.
- ***Virtual Desktop Interface (VDI)***: Many users believe VDI offers much stronger security than it actually does; common misconceptions include the notion that hackers cannot launch an attack into a virtual session, since no data are stored locally on an endpoint device and that ending a session (in a non-persistent VDI) sanitizes any threats present in that session. Once an adversary breaches or takes control of a node, they can potentially compromise the application underlying the workload on the VDI or even get a foot in the door to the enterprise network and data center. VDI has three weak spots:
  1. ***Exfiltration**:* VDI desktops typically have fast (10GB+) access to network resources, including internal file shares and databases. As these desktops are non-persistent and are randomly assigned at logon, it can be harder to track and record the data exfiltration, as it might be occurring across multiple desktops, IPs, and user accounts, rather than a single compromised workstation.
  2. ***Persistence**:* Malware can leverage a user’s roaming profile or a mapped network drive to allow itself to persist across reboots by reloading itself each time a user logs back in to his desktop.
  3. ***Exploitation**:* Non-persistent VDI does little to actually prevent exploitation from occurring. The only requirement limiting exploitation is that the initial exploit has to occur within the life of the VDI session before the desktop is rebooted.

## 4. DREAD Threat Model

The DREAD threat model is a form of quantitative risk analysis that involves rating the severity of a cyber threat; it allows organizations to look at security in a structured way, enabling them to analyze and identify every possible threat. Using the DREAD model [48], an organization can effectively prioritize threats in terms of mitigation by determining how much damage the threat has already caused and will cause in the future.

For each threat, the DREAD model considers the following five key points:

- ***Damage potential***: How great is the damage to the assets?
- ***Reproducibility***: How easy is it to reproduce and replicate the attack?
- ***Exploitability***: How much time and energy are required to exploit the threat?
- ***Affected users***: How many people, either inside or outside of the business, will be affected by the cyber threat?
- ***Discoverability***: How easy is it to discover the cyber threat?

By assessing a threat, all of the key considerations mentioned above must be taken into account and rated between 1 and 3: A rating of one indicates a low risk, a rating of two indicates a moderate risk, and a rating of three indicates a high risk. Thus, a threat will receive a total rating of between five and fifteen, and the total severity is as follows:

- ***Five to seven***: Low-risk threats.
- ***Eight to eleven***: Medium-risk threats.
- ***Twelve to fifteen***: High-risk threats.

Table 1 contains descriptions of the DREAD model’s five key points according to their levels of severity.

As shown in the Appendix A, we also divided the threats in the work-from-home culture into three categories: top-priority threats (see Table A1), high-priority threats (see Table A2), and low-priority threats (see Table A3). Please note that the DREAD key points were assigned a rating based on a discussion with subject matter experts from academia and industry. These ratings may change depending on the organization’s sector, how well the organization is protected from threats, and when the evaluation took place.

## 5. Threat Mitigation

Cyber-security risk mitigation involves applying different policies and processes in order to reduce the risk of a variety of threats. There are four types of risk mitigation: risk acceptance, risk avoidance, risk limitation, and risk transference.

- ***Risk acceptance***: Although risk acceptance does not reduce the effects of an attack, it is still considered a strategy. This strategy is a common option when the cost of other risk management options such as avoidance or limitation may outweigh the cost of the risk itself. A company that does not want to spend much money on avoiding risks that do not have high likelihoods of occurring will use the risk acceptance strategy.
- ***Risk avoidance***: Risk avoidance is the opposite of risk acceptance. In this case, any exposure to the risk is avoided. It is important to note that risk avoidance is usually the most expensive of all risk mitigation options.
- ***Risk limitation***: Risk limitation is the most common risk management strategy used by companies. This strategy limits a company’s exposure by taking some action. It employs a bit of risk acceptance and risk avoidance in some combination of the two. An example of the use of this strategy is when a company accepts that a disk drive may fail but aims to prevent a long down time if that occurs by having backups.
- ***Risk transference***: Risk transference is a strategy in which the risk is passed to a willing third party. For example, numerous companies outsource certain operations such as customer service and payroll services. This can be beneficial for a company when the area involved and potentially at risk is not one of its core competencies. This strategy is also used to enable the company to focus more on its core competencies.

**Figure 3:**
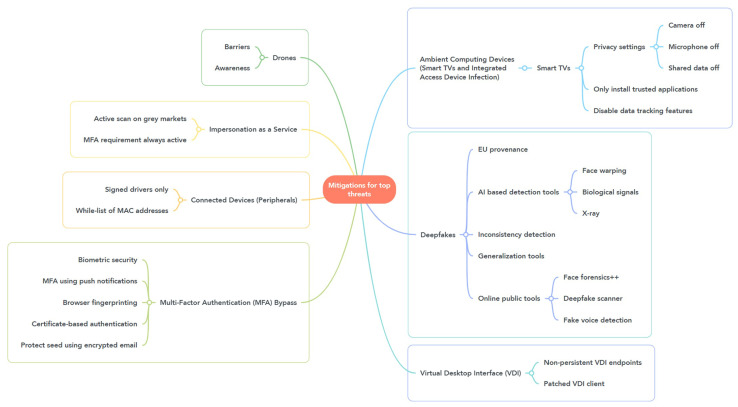
Mitigations for the top threats.

This section describes various methods that can be used to mitigate cyber threats. Figure 3 presents mitigations for the top threats.

### 5.1. Drones

Advanced techniques [49,50] have been proposed for the detection of drones that perform reconnaissance and violate privacy, but the proposed techniques are not practical for the protection of every employee. However, there are measures that can be taken to reduce the risk, such as using physical barriers—including walls or fences to restrict drone access—or using signal jamming devices or software to disrupt the drone’s communication system. It is important to note that signal jamming may be illegal in some areas and could interfere with other wireless communication devices. Additionally, reporting any suspicious drone activity to the authorities and keeping a watchful eye on the sky can also help prevent unauthorized reconnaissance.

### 5.2. Deepfakes

As deepfakes evolve and become more sophisticated and widely used, researchers have started to develop mechanisms capable of identifying deepfake media. Solutions have emerged from both industry and academia, which have proposed frameworks for authenticating the provenance of any media file on the Internet; these frameworks limit the propagation of misinformation, which is one of the motivations of adversaries using deepfakes. Existing solutions that can help in either limiting the damage caused by deepfake media or identifying them are presented below.

- ***Authentication of media via provenance***: Microsoft proposed the “AMP” (authentication of media via provenance) framework [51] to ensure the authentication of media by certifying provenance. The framework allows publishers to create signed metadata for a media instance. This metadata are stored centrally in a database by a trusted authority that can be queried by browsers and applications to check the media’s authenticity. The media’s authenticity can be communicated to the user via visual elements in the browser, indicating that an AMP manifest has been successfully located and verified. Similar to this is a framework known as "PROVENANCE" [52], which aims to help by warning users when the content they are looking at may be misinformation or disinformation. The PROVENANCE browser plugin checks the content that users see on the Internet and social media and provides warnings in their browser or social media feed regarding the authenticity of the media. In the future (when widely adopted), these frameworks will help build trust among viewers regarding the content and counter deeepfakes used to spread misinformation.
- ***Artifact-based detection***: Deepfakes often generate artifacts that are difficult for humans to detect. Researchers have proposed a few techniques that use ML and AI to identify those inconsistencies and detect deepfakes. One such technique is based on the observation that current deepfake algorithms only generate images of limited resolution, which need to be further warped to match the original faces extracted from the source video frames. The warping techniques leave distinctive artifacts in the resulting deepfake, which can be adequately captured by convolutional neural networks (CNNs) [53]. Similarly, in a paper titled "FakeCatcher" [54], the researchers’ key assertion is that biological signals hidden in portrait videos can be used as implicit descriptors of authenticity, because they are neither spatially nor temporally preserved in fake content; various biological signals, such as a heartbeat, pulse, and blood volume patterns hidden in portrait videos, were used to verify authenticity. In another study, the authors released an “in the wild” dataset http://cs.binghamton.edu/~ncilsal2/DeepFakesDataset/ (accessed on 21 June 2022) of fake portrait videos that they collected as a part of their experiment. The authors proposed a face X-ray technique for the detection of forgery in face images. The face X-ray of an input face image is a grayscale image that reveals whether the input image can be decomposed in the blending of two images from different sources. They showed the blending boundary for a forged image and the absence of blending boundary for a real image. The algorithm for computing a face X-ray can be trained without fake images generated by state-of-the-art face manipulation methods. The proposed method remains effective when applied to forgery performed by unseen face-manipulation techniques [55].
- ***Inconsistency-based detection***: Several techniques for identifying inconsistencies in media can be used for deepfake detection. Inconsistencies between audio speech patterns and mouth motion, speaker features, and visual facial features (e.g., a voice change but no face change) can help achieve the confidence score required for deepfake detection. In another study [56], the authors were able to detect manipulations of video by searching for and combining the evidence of multiple types of inconsistencies between the audio and visual channels: inconsistencies among the type of scenes detected in the audio and visual modalities (e.g., audio indoors, small room versus visual outdoors, and urban) and inconsistencies in speaker identity tracking over a video given audio-speaker features and visual face features (e.g., a voice change without any face changes). A temporal-aware pipeline for the automatic detection of deepfake videos was proposed [57], in which an algorithm leverages a CNN to extract frame-level object features. These features are used to train a recurrent neural network (RNN) that learns to classify by finding temporal inconsistencies that indicate if a video has been subject to manipulation or not.
- ***Semantic detection***: Algorithmic detection techniques that rely on statistical fingerprints and anomalies can be fooled with limited additional resources (e.g., algorithm development, data, or computing power). In the current media generation, deepfakes rely heavily on data-driven approaches, so they are prone to making semantic errors. Given this, a forensic technique was proposed in which facial expressions and movements that reflect an individual’s speaking pattern are modeled for deepfake detection [58]. Although not visually apparent, these correlations are often violated given the way that deepfake videos are created.

Along with the above approaches, other studies focused on the forensics of the deepfake content. In one such study, the authors demonstrated that each GAN (generative adversarial network) leaves its specific fingerprint in the images it generates, just as real-world cameras leave acquired images with traces of their photo-response non-uniformity patterns [59]. In other research, the focus was on developing the generalization ability of forensic models to detect new types of GAN images. The authors proposed using preprocessed images to train a forensic CNN model. By applying similar image-level preprocessing steps to both real and fake images, unstable low-level noise cues were destroyed, forcing the forensic model to learn more intrinsic features to classify the fake and real face images [60]. GANs are involved in generating a large amount of deepfake content; therefore, such approaches can prove vital in performing forensics after an incident has occurred.

There are also publicly available tools https://github.com/deepware/deepfake-scanner (accessed on 21 June 2022), https://github.com/dessa-oss/fake-voice-detection (accessed on 21 June 2022) and services https://deepware.ai/ (accessed on 21 June 2022) that help with detecting deepfake content.

### 5.3. Multi-Factor Authentication Bypass

A user’s account is more secure when MFA is enabled but it is not hermetically secure. MFA measures can be bypassed by using targeted attacks (e.g., SIM-SWAP attack) in order to obtain the user’s credentials for further exploitation. In addition, many services allow users to denote their devices as "trusted", thereby requiring fewer authentication measures. In order to avoid SIM-based attacks, there are physical-component-based MFA (e.g., RSA SecureID) or application-based MFA techniques (e.g., push notifications).

In order improve the security of MFA and prevent an attacker from recalculating the current key, it is extremely important to maintain the confidentiality of the initial secret (seed) used to initialize the component/application. To prevent the reuse of primary values, the seed must be transmitted in an encrypted manner that cannot be reproduced (e.g., sent by encrypted mail using Gmail’s confidential mode).

Several works in the field of MFA have examined existing solutions to study their advantages and disadvantages. Matt et al. [61] provided a broad overview of the different MFA implementations, which showed that most implementations are not useful in a situation in which the endpoint device is infected. Although USB key implementation (e.g., YubiKey [62]) is more complex, it may make it difficult for an attacker to overcome the security layer.

### 5.4. Smart TV Data Leakage

Smart TV platforms are the latest IoT devices found to be “spying” on users and leaking sensitive data to companies and manufacturers. Ren et al. [63] showed that smart TVs collect and pass on information about users’ viewing habits and preferences to partner companies. In the same way, an adversary could gather information about a user’s habits or exploit the camera and microphone installed on these smart accessories to gather information or perform extortion or espionage. In order to prevent and reduce the risk of data leakage, there are several steps that a user can take:

- ***Privacy setting***: Turn off the camera and microphone in the TV’s settings.
- ***Trusted applications***: Only use trusted applications from the original application store.
- ***Data collection***: Disable data collection from third parties (e.g., LG LivePlus service, Samsung viewing information services, Vizio viewing data services, and TCL/Roku information from other inputs.

### 5.5. Impersonation as a Service

To prevent the impersonation of a digital identity on the Internet, it is important to use strong authentication methods such as MFA and digital certificates. These methods provide an additional layer of security to verify the identity of the user. It is also important to regularly monitor and review access logs to detect any unauthorized access attempts. Additionally, educating users on the importance of creating strong passwords, avoiding phishing attacks, and regularly updating their software can also help prevent impersonation. Finally, implementing a secure and robust identity management system can help organizations ensure the safety of their users’ digital identities.

### 5.6. Peripheral Infection

Interference with peripherals requires an adversary with experience and many resources. These attacks require access to the source code, a company’s servers, or a product’s supply chain. To avoid infection by an illegitimate driver, Microsoft added a feature that is enabled by default and prevents the execution of digitally signed drivers. The security level can also be increased by creating an "allow-list" of authorized external connections. Hessam et al. [64] suggested an allow-list-based defense technique to determine which USB plugins are trusted, using different features (e.g., product ID, vendor ID, and firmware revision). Today, there are solutions that large companies can use to manage white-listing, e.g., Microsoft’s Intune "restrict USB devices" templates [65].

### 5.7. Exploit Virtual Desktop Infrastructure

Virtual desktop infrastructure (VDI) refers to hosting desktop environments on a central server. VDI is a type of desktop virtualization, as the specific desktop images run on virtual machines (VMs) and are delivered to end clients over a network. There are two types of VDI implementation: persistent VDI and non-persistent VDI.

- ***Persistent VDI***: the user always logs into the same desktop image, with all changes to applications and data retained.
- ***Non-persistent VDI***: in this type, no changes are saved; a clean image is loaded in every connection.

In order to prevent an adversary from interfering in the traffic of a VDI session, the non-persistent VDI platform enables the sanitization of any threat present in a session. Therefore, managing a non-persistent VDI environment will make it difficult for an attacker to conduct a continuous attack and will force the attacker to perform the initial entry process each time and start from the entry point. Using centralized implementation and maintaining an updated and hardened image (Golden VDI image) to initialize a VDI session will make it challenging for an attacker to exploit various vulnerabilities.

### 5.8. Network Misconfigurations

Router configuration errors can occur unintentionally or due to a lack of understanding of the network structure and the various protocols. There are different types of attacks that can be performed on ports that are needlessly accessible. Several preventive methods can help prevent misconfiguration:

- ***Restrict access***: Restrict access to endpoints using an operating system firewall (e.g., Windows defender firewall or anti-virus firewalls).
- ***Virtual patching***: Implement a web application firewall (WAF), which is an application that protects web applications from a variety of application layer attacks, such as cross-site scripting (XSS), SQL injection, and cookie poisoning. Virtual patching refers to the rapid development and short-term implementation of a security policy meant to prevent an exploit from occurring as a result of a newly discovered vulnerability. Virtual patching is usually supplied by WAFs.
- ***Configuration monitor***: Use home monitor configuration services that check the router configuration template (e.g., F-Secure router checker) or automatic template inference, as suggested by Kakarla et al. [66]

### 5.9. Phishing

The most effective measure against phishing is to increase employees’ awareness regarding the different ways of identifying phishing emails—for example, emails that provide links to unknown sites that ask employees to provide personal data, emails from suspicious email addresses, and emails in which the language quality is poor, with generic salutations, a suspicious attachment, and/or a false sense of urgency. To create such awareness, the company can implement an awareness program in which phishing emails are sent to employees to assess and create awareness. Such campaigns can be run using tools such as GoPhish https://getgophish.com/ (accessed on 21 June 2022) and Cofense https://cofense.com/product-services/phishme/ (accessed on 21 June 2022). In cases in which the emails received by employees become more prevalent or sophisticated, advanced phishing detection tools can be employed by the company, using solutions from Check Point (https://www.checkpoint.com/harmony/email-security/email-office/ (accessed on 21 June 2022)), (https://www.avanan.com/anti-phishing-software (accessed on 21 June 2022)), Brandshield (https://www.brandshield.com/products/anti-phishing/ (accessed on 21 June 2022)), (https://www.barracuda.com/products/email-protection/phishing-protection (accessed on 21 June 2022)), and others. In addition to these commercial tools, the company can develop in-house AI-based solutions [67,68,69].

### 5.10. DoS on Both Network and Application Services (Amplification/Reflection Methods)

In addition to existing gateway-level solutions for the corporate network, there is a need to focus on endpoint devices by analyzing the traffic coming out of these devices through the corporate gateway and another to prevent the company’s laptops from being used to perform DoS and DDoS attacks. The company must ensure that the traffic originating from the endpoints (e.g., corporate laptops) is consistent in terms of the source IP addresses and is not being spoofed, as inconsistency is a strong indication that a corporate laptop is being used to perform a DoS or DDoS attack. There is also a need to detect cases in which there is a heavy flow of broadcasted messages aimed at exhausting the network capabilities of the other connected devices on the same network. To boost existing DoS detection capabilities, technical teams can develop in-house, ML-based DoS and DDoS solutions [70,71].

### 5.11. Malware Infection

The number of malware attack vectors is greater in the work-from-home environment, where the use of unsecured external devices, such as Bluetooth smartwatches, home assistant devices, wireless keyboard/mice, and USB drives, is less controlled than in the corporate setting. Therefore, employees should be made aware of the threats posed by external devices and encouraged to adopt safe practices such as disconnecting from external media such as Bluetooth and NFC devices when they are not in use. Additionally, if required, companies can prohibit employees from connecting such external devices to corporate laptops; employees should also be trained to only download and install software from trusted sources and avoid installing pirated software.

### 5.12. Identity Theft (Identity Fraud/Account)

Awareness regarding the misuse of adversarial AI should be promoted among employees by presenting case studies that illustrate how adversaries can use adversarial AI to perform identity theft and trick employees. Additionally, to assess employees’ security awareness levels, the company should perform campaigns similar to the phishing campaigns previously mentioned. Regarding the identity theft of users based on impersonating credentials, the company should monitor the dark web for the company’s sensitive data or the data of their key employees so that it can raise timely alerts and avoid any serious issues. Websites that report news on email dumps (e.g., haveibeenpwned https://haveibeenpwned.com/ (accessed on 21 June 2022) and pastebin https://pastebin.com/ (accessed on 21 June 2022)) should also be monitored. An alert is raised if the records of employees are found, as there is a good chance that an employee may have used the same password for multiple accounts.

### 5.13. Exploitation of Wireless Communication Protocols

A strict policy should be in place to avoid connecting the corporate laptop to insecure Wi-Fi (public Wi-Fi, Wi-Fi using weak algorithms, etc.). To improve security, the use of a VPN should be enforced. A detailed document instructing employees how to securely configure their home routers should be provided, and the technical support team should provide the necessary support to configure routers, if required. Awareness of the need to turn off unwanted wireless media, such as Bluetooth and NFC devices, should be promoted among employees.

### 5.14. Infected Trusted Software/Mobile Applications

Proper privilege management can reduce the damage caused by installing insecure applications on corporate laptops. Various tools that support privilege management are available, including BeyondTrust’s privilege management tool, which provides features such as trusted application protection (TAP) that issue an alert when an untrusted application is executed and blocks its execution [72]. The signatures of the applications that the user installs should be validated, and only applications signed by trusted entities should be permitted to be installed.

### 5.15. Man-in-the-Middle (MiTM)/Session Hijacking and Fake SSL Certificates

A VPN should be used to prevent MiTM attacks; since most of the traffic is encrypted before leaving the laptop, a VPN prevents the leakage of any sensitive information. Agents that are installed on end devices (e.g., laptops) can analyze inbound and outbound traffic to detect MiTM attacks. For example, companies offer services aimed at the detection of MiTM attacks and mitigation against MiTM attacks (e.g., Check Point’s Sandblast mobile application https://www.checkpoint.com/downloads/products/sandblast-mobile-onp-ds.pdf (accessed on 21 June 2022), which is an "on-device" network protection application [73]). In one study, the authors proposed a novel portable method based on analyzing the ICMP echo response, using an autoencoder to identify MiTM attacks [74]; however, the use of such a method in a work-from-home environment has not been examined. Any SSL communication certificates should be validated by both the company’s server and the endpoints (corporate laptops) to avoid the use of fake certificates generated by an adversary.

### 5.16. Side-Channel Attacks

VPNs often use data compression to provide better network performance, but that can lead to compression-based side-channel attacks. Therefore, companies must verify that the VPN employed does not leak any information by compressing transmitted data that an adversary can infer. In [75], the author showed that a TCP-based VPN is not vulnerable to compression-based side-channel attacks, since the patterns of the traffic cannot be identified, making this type of VPN a good choice. Alternatively, an existing VPN can be configured so that compression-based side-channel attacks can be prevented.

### 5.17. Obsolescence

Proper standard operating procedures (SOPs) can help employees perform the steps required to keep their systems updated and avoid problems arising from an obsolete system. Multiple reminders can be sent to employees to encourage them to upgrade outdated applications/OSs.

### 5.18. Network Reconnaissance, Network Traffic Manipulation, and Information Gathering

Employees should be instructed to keep home IoT devices away from their work-from-home environments, and companies should explain the risks posed by having such devices in close proximity. Companies should also stress the need to keep home IoT devices updated and avoid the use of default credentials to maintain the security of corporate data and employees’ privacy. Companies can consider employing tools similar to Okyo Garde https://www.paloaltonetworks.com/okyo (accessed on 21 June 2022) (a product of Palo Alto Networks), which enhance the home network security of employees that have access to highly confidential information. The use of end device protection agents that issue alerts when there is an illegitimate connection within the local network can further enhance the overall security and provide protection from threats associated with local IoT devices.

### 5.19. Eavesdropping, Interception, Hijacking

In order to avoid unintentional information disclosure, companies must reduce the use of plaintext communication and utilize protocols for communication encryption. This can be accomplished by using private and secured networks such as VPNs and by implementing and enforcing security policies, (e.g., utilizing encrypted DNS queries (DoT-DNS over TLS and DoH-DNS over HTTPS), which are two standards developed for encrypting plaintext DNS traffic [76]) or setting browsers with rigorous settings to allow only encrypted traffic (permitting HTTPS as opposed to HTTP) or plaintext communication is blocked using firewalls.

In order to protect their Wi-Fi network, companies should make sure that their routers support WPA3 (Wi-Fi Protected Access 3), which represents the latest generation in mainstream security for wireless networks, and that the default management interface passwords set by manufacturers for the routers are replaced.

### 5.20. Credential Discovery

In order to avoid the theft of crucial information from endpoints, a number of settings can be hardened, which will make it difficult for attackers to access information.

- Disable the WDigest store credentials in the memory (legacy challenge/response protocol) using the *SecurityProviders* registry key.
- Ensure that both LM and NTLMv1 are disabled using the *Local Policies/Security Options* registry key.
- Set the Local Security Authority Subsystem Service (LSASS) to protected mode.
- Limit credential caching using the *SECURITY/Cache* registry key.

### 5.21. Cryptanalysis

Several tools can be used to prevent cryptanalysis, such as encryption software, which uses strong cryptographic algorithms to secure data; key management software, which securely stores and manages encryption keys; two-factor authentication, which requires users to provide two forms of authentication to access a system; and digital certificates, which verify the identity of a user or system and establish a secure connection between them. Digital certificates are commonly used in SSL/TLS encryption protocols for secure web browsing. These tools help to prevent unauthorized access to systems and data, including encryption keys, and ensure that data have not been tampered with when they are in transit.

### 5.22. Physical Attack (Deliberate or Intentional)

In order to reduce the risk associated with lost or stolen physical devices, a company can use a disk encryption mechanism, e.g., BitLocker (https://docs.microsoft.com/en-us/windows/security/information-protection/bitlocker/bitlocker-overview, accessed on 21 June 2022) or software (e.g., drivestrike https://drivestrike.com/features/remote-lock/ (accessed on 21 June 2022)). that allows full control and remote locking of the computer or deletion of the information it contains. Companies should also invest effort into increasing employees’ awareness about the need to prevent data leaks and the importance of using complex passwords and encryption mechanisms; in addition, companies should prevent employees from physically taking documents out of the company’s buildings.

### 5.23. Physical Attack (Unintentional Damage or Loss of Information or IT Assets)

Performing remote backups of valuable data (e.g., employees’ laptops) on the company’s servers can prevent the loss of information. Backups of the data on employees’ devices can be performed using dedicated software or Microsoft’s Group Policy Object (GPO https://docs.microsoft.com/en-us/previous-versions/windows/desktop/policy/group-policy-objects (accessed on 21 June 2022)) rules.

## 6. Discussion

The necessity of remote work during the COVID-19 pandemic created new information security concerns. The work-from-home culture did not cease as the pandemic waned, and organizations are increasingly adopting this approach. To address the security issues present in work-from-home environments, there is a need to create asset and threat taxonomies unique to the work-from-home culture. In this paper, we provided the necessary taxonomies and presented the findings of our in-depth risk analysis, in which each threat’s effects and means of mitigation were examined. Our threat and asset analysis revealed a range of threats that could potentially compromise a company’s corporate networks through a variety of new attack vectors that are present in work-from-home environments. Therefore, it is critical for companies to carefully consider the remote-work approach and the privileges granted to employees in this setting; install appropriate protection products; and establish policies aimed at mitigating these risks. It is important to note that the taxonomy needs to be updated over time as new threats emerge and others become obsolete.

In addition, the risk scores may differ for each organization, depending on the organization’s preparedness for specific threats, the overall risk to the organization’s sector, and the roles of the employees. As a result, future research could be aimed at quantifying the increased risk associated with the adoption of the work-from-home culture; the ability to quantify this risk would enable organizations considering switching from the traditional work culture to the work-from-home culture to analyze the costs and benefits of doing so from a risk management perspective and institute policies aimed at mitigating the risks, prior to adopting the new culture.

## Figures and Tables

**Table 1:** Descriptions of the DREAD model’s key points.

Rating	Low (1)	Medium (2)	High (3)
**Damage potential**	The attacker subverts the system and can inflict minor damage.	The attacker subverts the system and can inflict moderate damage.	The attacker subverts the system and can inflict serious damage.
**Reproducibility**	The attack is very difficult to reproduce, even with full knowledge of the security hole.	The attack can be reproduced but only in limited settings.	The attack can be reproduced every time.
**Exploitability**	The attack requires an extremely skilled operator with in-depth knowledge of the system in order to exploit it.	The attack requires a skilled operator with fundamental knowledge of the system in order to exploit it.	The attack requires little or no knowledge of the system in order to exploit it.
**Affected users**	A very small percentage of everyday users will be affected by the attack.	A good-sized portion of everyday users will be affected by the attack.	The majority of everyday users will be affected by the attack.
**Discoverability**	Vulnerabilities are difficult to find and, if found, are very difficult to weaponize. It is extremely difficult to attack applications and systems.	Vulnerabilities are not common and are found only in certain applications and systems. The attack requires specific skills to discover exploitable weaknesses.	Published information readily explains the attack. Vulnerabilities are found in the most commonly used applications and systems.

## Appendix A

**Table A1:** Top threats and use cases.

Threat Categories	Possible Scenarios/Attack Vectors	D	R	E	A	D	DREAD Rating	Time Horizon
Drones	A small computer, such as a Raspberry Pi, could be dropped in the vicinity of a victim’s building using a drone. It could mimic a Wi-Fi network in order to steal data from tablets and smartphones, or hijack Bluetooth peripherals, such as mice and keyboards. Keylogging would enable a drone-mounted computer to steal passwords from users.	2	3	2	1	2	Medium	1–3 years
	Drones equipped with a software-defined radio located near a TV antenna can transmit a signal that is more powerful than the one broadcasted by legitimate TV networks, overriding the legitimate signal and displaying their desired video on the TV. It can lead to phishing attacks.	1	2	1	1	2	Low	1–3 years
	Drones can fly into the range of the victim’s computer such that it can mimic a fake wireless printer and obtain the file that a victim intended to print.	1	2	1	1	2	Low	1–3 years
	Drones can be used to drop malicious thumb drives in the home of the victim so that when the thumb drive is plugged into the personal computer/laptop, it infect the computer, leading to the adversary’s presence on the home network.	3	3	2	2	2	High	1–3 years
	Drones can also be used to record the intimate moments of the victim which can help the adversary blackmail the victim.	2	2	2	1	2	Medium	1–3 years
	Drones can fly near the victim’s window to monitor a screen/TV containing confidential data.	2	2	2	1	2	Medium	1–3 years
Deepfakes	An adversary can make a synthetic video of a senior manager asking an employee about sensitive information.	3	3	2	2	3	High	1–3 years
	Call-based spear phishing attacks can be enhanced using real-time deepfakes of someone whom the victim trusts. For text-based phishing, tweets and emails can be generated to attract a specific victim, or style transfer techniques can be used to mimic a colleague. Similar approaches can evade email spam filters.	2	3	2	2	3	High	1–3 years
	Deepfakes can be used to make a synthetic video of a key person that can be played on a online meeting platform, firing their employees or playing it before a crucial event to disrupt it, similar to what occurred to the CEO of better.com.	2	2	2	1	3	Medium	1–3 years
	A synthetic video of the victim employee could be made and shared with his/her senior manager by another employee to foster distrust among them.	1	2	2	1	3	Medium	1–3 years
	The CEO or any employee can send synthetic voice mail over email asking employees to donate to his/her charity.	3	3	2	2	3	High	1–3 years
	A synthetic video of a key person could be made and published on the Internet in which the speaker is speaking poorly about the company’s policies, announcing fake offers, etc.	2	2	2	2	3	Medium	1–3 years
	An adversary can create fake images/videos of the victim employee in a compromising positions, drinking, smoking, or even naked. Thus, the victim employee can be blackmailed.	3	2	2	1	3	Medium	1–3 years
	An adversary can use deepfake technology to steal the identity of a deceased person for financial gain. An adversary can open a new account using the identity of a deceased person by using a synthetic video in the online verification process.	1	1	1	1	1	Low	1–3 years
	An adversary can make a fraudulent insurance or other claim on behalf of a deceased person. Claims can successfully continue to be made on pensions, life insurance, and benefits for many years after a person dies; this could be done either by a family member or professional fraudster. Deepfakes could be used to persuade an official that a customer is still alive.	1	1	1	1	1	Low	1–3 years
	An insider adversary can create relevant fake accounting transactions, i.e., journal entries of the ‘Enterprise Resource Planning’ (ERP) systems to evade anomaly detection in an audit performed by ‘Computer Assisted Audit Techniques’ (CAATs).	1	1	1	1	1	Low	1–3 years
	AI can be used bypass the fingerprint locks of secured mobile phones/laptops by generating a masterprint that helps the adversary unlock the devices to gain access to critical information.	2	1	1	1	2	Low	1–3 years
	Adversaries can build fake personas on online social networks (OSNs) to connect with their targets. To evade fake profile detectors, a profile can be cloned and slightly altered using AI so that the fake profiles will appear different yet reflect the same personality. The adversary can then use a number of AI techniques to alter or mask the photos from detection. To build connections, a link prediction model can be used to maximize the acceptance rate, and a deep-learning-based Chabot can be used to maintain conversations with the fake profiles.	2	2	1	1	1	Low	3–5 years
Multi-Factor Authentication (MFA) Bypass	An adversary can use the credentials and soft token from the stolen laptop of victim A to connect to a VPN and then use the credentials of victim B to log into other services thus bypassing MFA for victim B.	3	2	2	1	2	Medium	Present
	An adversary could gain access to the victim’s email and discover the MFA URL responsible for pairing the MFA soft token of a victim’s phone with the victim’s user identity in the company’s MFA server. The MFA URL obtained could contain a cryptographic seed that can be leveraged by the adversary to generate OTP codes after breaking the PIN and using the timestamp with it.	2	2	1	1	1	Low	Present
	Having access to the victim’s laptop, an adversary can exploit DPAPI, i.e., the Windows API responsible for encryption and decryption of the credentials, to obtain the domain backup key that is capable of decrypting an encrypted blob and hence can escalate the attack to other users in the domain.	3	2	1	3	2	Medium	Present
	An adversary can exploit other ways of connecting to critical servers (like SMB, RPC, etc.) to bypass MFA.	2	2	2	1	2	Medium	Present
	An adversary can fool a telecom company in order to get a SIM and steal the employee’s number, performing a SIM SWAP attack.	2	2	2	1	2	Medium	Present
	An adversary can take advantage of an existing trusted connection to hijack the session token and re-use it without authentication.	2	2	2	1	1	Medium	Present
Ambient Computing Devices (Smart TVs and Integrated Access Device Infection)	An adversary can leverage a compromised mobile device of the victim placed near the keyboard to recover the keystrokes that were typed on the keyboard. A similar threat may arise from other IoT devices.	2	2	1	1	2	Medium	3–5 years
	An adversary can use an approach that can recover the keystrokes from the victim’s eye movements. The attack’s likelihood of success increases in home offices where employees use the camera of a laptop or connect the screen to smart TVs that have a built-in camera.	2	2	1	1	2	Medium	3–5 years
	Compromised IoT devices present on the home network can aid in performing attacks that can be performed on the local network like ARP poisoning, sniffing, DNS poisoning, etc.	2	2	2	1	2	Medium	Present
	An adversary can use smart TVs in order to capture data presented on the TV screen (i.e., a Teams meeting).	2	2	2	1	2	Medium	1–3 years
	An adversary can control a smart TV camera/microphone to record the home environment.	2	2	2	1	2	Medium	1–3 years
	An adversary can control the screen content in order to provide fake content or execute phishing attacks.	1	1	2	1	1	Low	1–3 years
	An adversary can use the TV as an entry point and pivot in the network or gather information about available services and open ports.	1	2	2	2	1	Low	1–3 years
	An adversary can steal sensitive data (i.e., cookies) from smart TV services.	1	2	2	2	1	Low	1–3 years
	An adversary can corrupt smart TV firmware in order to take control of the TV.	2	2	2	1	1	Medium	3–5 years
	An adversary can craft a DVB signal to take control of the TV browser application.	1	2	2	1	1	Low	3–5 years
	An adversary can take control of smart streamers and infect connected PCs and smart TVs using a Bluetooth connection.	2	2	2	1	1	Medium	1–3 years
	An adversary can use smart streamers to record the environment using a built-in microphone.	2	2	2	1	1	Medium	1–3 years
Impersonation as a Service	An adversary can purchase the profile of the targeted employee to gain his/her profile and perform certain activities or avoid MFA.	2	2	3	2	2	Low	Present
Connected Devices (Peripherals)	An adversary can infect a peripheral device driver with a malicious payload.	2	2	2	1	1	Medium	1–3 years
	An adversary can replace the peripheral device’s firmware.	2	2	2	1	1	Medium	1–3 years
	An adversary can utilize a peripheral device to perform data extraction.	2	2	1	2	1	Medium	3–5 years
Virtual Desktop Interface (VDI)	An adversary can hijack a remote session to the VDI console.	2	2	1	1	1	Low	1–3 years
	An adversary can spread within the network from the VDI console.	3	1	1	2	1	Medium	1–3 years

**Table A2:** High-priority threats and use cases.

Threat Categories	Threats	Possible Scenarios/Attack Vectors	Impacted Devices	D	R	E	A	D	DREAD Rating	Time Horizon
Nefarious Activity/ Abuse	Phishing	Phishing emails regarding an online meeting or some new offer introduced by the company can be used to lure an employee.	Company’s laptop	2	2	2	1	1	Medium	Present
		Based on network activity reconnaissance, spear phishing emails can be crafted in a more effective way.	Digital devices connected to home router, home router	2	2	2	1	1	Medium	1–3 years
	DoS on both network and application services (amplification/reflection methods)	An adversary present on the employee’s home Wi-Fi network can flood the remote machine and can cause a DoS attack.	Company’s laptop, home router Wi-Fi segment	1	1	2	1	1	Low	Present
	Malware infection	Infection can occur from an external device plugged into the employee’s PC (e.g., a smartwatch, etc.).	Company’s laptop	2	2	1	1	2	Medium	1–3 years
		An advanced phishing attack performed via email may infect an employee’s PC (e.g., asking an employee to download updated video conferencing software).	Company’s laptop	2	2	2	1	1	Medium	Present
	Identity theft (identity fraud/account)	An adversary can impersonate an employee’s manager and ask the employee to pass on certain confidential information. ML can be used to generate the synthetic voice of the manager.	-	3	3	2	2	3	High	1–3 years
		An adversary can purchase the profile of a targeted employee to obtain his/her profile to perform certain activities or to avoid enabling MFA.	-	1	1	2	1	1	Low	1–3 years
	Exploitation of wireless communication protocols	An adversary can remotely monitor an NFC connection.	Company’s laptop	1	2	1	1	1	Low	1–3 years
		An adversary can remotely control and spread the components in the network using a malicious Bluetooth connection.	Digital devices connected to home router, home router, company’s laptop	2	2	1	1	1	Low	1–3 years
		An adversary can crack Wi-Fi passwords using bruteforce or KRACK exploits and monitor network activities.	Home router	1	2	2	1	2	Medium	Present
	Infected trusted software/mobile applications	An adversary can reverse engineer an existing app of the company and float a version containing malicious code.	Personal/company’s phone	2	2	1	3	2	Medium	Present
Eavesdropping/ interception/ hijacking	Man-in-the-middle (MiTM)/session hijacking	An adversary can set up an MiTM box to sniff and actively alter data accessed by the victim; this can be relevant for mobile traffic if not applicable on a laptop due to proxy tunneling.	Digital devices connected to home router, home router, IoT devices	2	2	1	1	2	Medium	Present
	Side-channel attacks	Side-channel attacks can be performed on the home router of the employee to understand the applications used by the employee and perform the targeted attack.	Company’s laptop, home router, IoT devices	1	2	1	1	1	Low	3–5 years
	Fake SSL certificates	Tools like BurpSuite can be used to inject a fake certificate that can help an adversary peek into the encrypted traffic (this attack also applies to the employee’s mobile phone); this can be relevant for mobile traffic if not applicable on a laptop due to proxy tunneling.	Digital devices connected to home router, home router	2	2	1	1	2	Medium	Present
Misconfiguration of Systems and Technologies	Obsolescence	If an employee is using old equipment, he/she may not be able to perform the required or recommended updates.	Company’s laptop	1	2	1	1	1	Low	Present
Smart Devices (IoT Devices)	Network reconnaissance, information gathering	An adversary can use a compromised camera to take photos of the employee and then blackmail him/her.	Digital devices connected to home router, home router, IoT devices	2	2	1	1	1	Low	1–3 years
		An adversary can use a compromised camera to map an employee’s keystrokes on the company’s laptop to obtain credentials or understand the communication taking place by email.	Digital devices connected to home router, home router, IoT devices	2	2	1	1	1	Low	1–3 years
		An adversary can use compromised IoTs to record voice and video conferences.	Digital devices connected to home router, home router, IoT devices	2	2	1	1	1	Low	1–3 years
		An adversary present on the home network can quickly check for weaknesses in the network and scan for vulnerable devices.	Digital devices connected to home router, home router, company’s laptop, IoT devices	2	1	2	1	2	Medium	Present

**Table A3:** Low-priority threats and use cases.

Threat Categories	Threats	Possible Scenarios/Attack Vectors	Impacted Devices	D	R	E	A	D	DREAD Rating	Time Horizon
Eavesdropping/ Interception/ Hijacking	Eavesdropping (DNS poisoning/DNS spoofing/DNS manipulation)	An adversary can use a compromised home router to sniff HTTP and DNS requests for reconnaissance; thus he/she can perform a targeted phishing attack (spear phishing) which will be aligned with the current actions of the employees.	Home router, Wi-Fi segment, IoT devices	2	2	1	1	2	Medium	Present
		Unsecured Wi-Fi can enable an adversary to perform reconnaissance and examine the network to identify the OS, applications, etc. running on the connected digital devices to target them.	Company’s laptop, home router, IoT devices	2	1	2	1	2	Medium	Present
	Side-channel attacks	Using the physical properties of a lamp in the home or the LED of a speaker, an adversary can record the conversation happening in a room in the house.	-	3	2	2	2	2	Medium	1–3 years
	Eavesdropping (network reconnaissance and information gathering)	An adversary can use DNS queries to identify the version of the OS or software installed on the device in order to use the most suitable CVEs.	Home router, IoT devices	1	2	2	1	1	Low	Present
		An adversary can break into the employee’s house when no one is home (as ascertained by cameras) and steal sensitive data and equipment.	Digital devices connected to home router, home router, IoT devices	1	2	3	1	1	Medium	1–3 years
	Corporate espionage	Home offices provide ample opportunities for the insider adversary to perform corporate espionage (e.g., extract data from a hard disk drive, record a meeting (at which sensitive information is discussed) on a mobile phone and share it with a competitor.	Company’s laptop, IoT devices	3	1	1	3	1	Medium	Present
Misconfiguration of Systems and Technologies	Credential discovery	Software can unintentionally reveal employee’s credentials (e.g., computer registry), or an adversary that has compromised a victim’s system can obtain the credentials in cleartext.	Company’s laptop	2	1	1	1	1	Low	Present
		An adversary can crack and obtain weak local administrator credentials by dumping lsass.exe (with software such as Mimi Katz) to obtain credentials in cleartext.	Company’s laptop	2	2	1	1	2	Medium	Present
	Other misconfigurations	An adversary can misconfigure an employee’s mobile phone to accept and run untrusted APKs in order to compromise the phone.	Personal/company’s phone	2	2	1	3	2	Medium	Present
		An employee could set his/her AV client to run with low privileges or to a limited folders, leaving it vulnerable to infection by malware.	Company’s laptop	1	2	1	1	1	Low	Present
		Changes made to an application or its configuration due to an employee’s lack of appropriate knowledge can create issues related to authorization or may leave a device in a vulnerable state.	Company’s laptop	1	2	1	1	1	Low	Present
Physical Attack (Deliberate/ Intentional)	Sabotage	Anyone with a grudge against a victim employee can intentionally destroy or damage digital devices or network communication to interfere with their normal functionality.	Company’s laptop, company’s documents, home router	1	2	2	1	1	Low	Present
	Vandalism	Anyone with a grudge against a victim employee can deliberately destroy or damage the company’s assets in the employee’s home or assets that are accessible via devices in the employee’s home.	Company’s laptop, company’s documents, home router	1	2	2	1	1	Low	Present
	Information leakage/sharing	An adversary can steal sensitive printed documents from an employee’s home.	Company’s documents	1	2	2	1	1	Low	Present
		An adversary can steal sensitive information, like passwords, from the RAM of a stolen laptop.	Company’s laptop	1	1	1	1	1	Low	Present
	Theft (devices, storage media, and documents)	Any thief can steal a company asset from an employee’s home for monitory gain.	Company’s laptop	1	2	2	1	1	Low	Present
Unintentional Damage/Loss of Information or IT Assets	Loss of storage media and documents	Children in the home can damage or destroy sensitive documents or devices owned by the company.	Company’s documents and removable devices	1	1	3	1	1	Low	Present
		An employee could lose his/her soft token or mobile phone hence might be unable to authenticate while using MFA method.	Personal/company’s mobile phone	1	1	1	1	1	Low	Present
	Damage caused by the employee or his/her family members	Liquid spilled on a company device in an employee’s home could damage the device.	Company’s laptop	1	1	3	1	1	Low	Present
	Maintenance errors	Disruption in the network or some other actions (performed by an employee or by background software services) might cause operational errors that could lead to a loss of information.	Company’s laptop	1	2	1	1	1	Low	Present
	Installation errors	Employees working at home may have a hard time installing an application, and it could be difficult for them to obtain remote assistance.	Company’s laptop	1	2	1	1	1	Low	Present
		Hardware failure in a digital device takes time to recover from in home offices.	Company’s laptop, home router	1	2	1	1	1	Low	Present

## References

[1] Gartner Survey 2020. https://www.gartner.com/en/newsroom/press-releases/2020-07-14-gartner-survey-reveals-82-percent-of-company-leaders-plan-to-allow-employees-to-work-remotely-some-of-the-time.

[2] Bitaab M., Cho H., Oest A., Zhang P., Sun Z., Pourmohamad R., Kim D., Bao T., Wang R., Shoshitaishvili Y. Scam pandemic: How attackers exploit public fear through phishing. Proceedings of the 2020 APWG Symposium on Electronic Crime Research (eCrime).

[3] CISA Understanding Denial-of-Service Attacks. https://www.cisa.gov/uscert/ncas/tips/ST04-015.

[4] Allot Glossary of Common DDoS Attacks. https://www.allot.com/ddos-attack-glossary/.

[5] Halavais A.C. (2001). The Slashdot Effect: Analysis of a Large-Scale Public Conversation on the World Wide Web.

[6] Jia Y., Zhang Y., Weiss R.J., Wang Q., Shen J., Ren F., Chen Z., Nguyen P., Pang R., Moreno I.L. (2018). Transfer learning from speaker verification to multispeaker text-to-speech synthesis. arXiv.

[7] Wiefling S., Iacono L.L., Dürmuth M. Is this really you? An empirical study on risk-based authentication applied in the wild. Proceedings of the IFIP International Conference on ICT Systems Security and Privacy Protection.

[8] Campobasso M., Allodi L. Impersonation-as-a-service: Characterizing the emerging criminal infrastructure for user impersonation at scale. Proceedings of the 2020 ACM SIGSAC Conference on Computer and Communications Security.

[9] IEEE Computer Society LAN MAN Standard Committee (1999). Wireless LAN Medium Access Control (MAC) and Physical Layer (PHY) Specifications. ANSI/IEEE Std. 802.11, 1999 Edition.

[10] Vanhoef M., Piessens F. Key reinstallation attacks: Forcing nonce reuse in WPA2. Proceedings of the 2017 ACM SIGSAC Conference on Computer and Communications Security.

[11] Heartfield R., Loukas G., Budimir S., Bezemskij A., Fontaine J.R., Filippoupolitis A., Roesch E. (2018). A taxonomy of cyber-physical threats and impact in the smart home. Comput. Secur..

[12] Seri B., Livne A. (2019). Exploiting Blueborne in Linux-Based Iot Devices.

[13] Kennedy T., Hunt R. A review of WPAN security: Attacks and prevention. Proceedings of the International Conference on Mobile Technology, Applications, and Systems.

[14] Wolff E.D., Growley K., Gruden M. (2021). Navigating the solarwinds supply chain attack. Procure. Lawyer.

[15] Razer’s Driver Infected. https://www.computerworld.com/article/2527857/gaming-mouse-maker-razer-hit-with-infected-firmware.html.

[16] Maskiewicz J., Ellis B., Mouradian J., Shacham H. Mouse trap: Exploiting firmware updates in {USB} peripherals. Proceedings of the 8th {USENIX} Workshop on Offensive Technologies ({WOOT} 14.

[17] Burgerking Havon on Google Assistant. https://www.engadget.com/2017-04-12-burger-king-wreaks-havoc-on-google-assistant-with-whopper-ad.html.

[18] Rogue Payment Warning—Alexa. https://www.telegraph.co.uk/news/2017/01/08/amazon-echo-rogue-payment-warning-tv-show-causes-alexa-order/.

[19] Pingle B., Mairaj A., Javaid A.Y. Real-world man-in-the-middle (MITM) attack implementation using open source tools for instructional use. Proceedings of the 2018 IEEE International Conference on Electro/Information Technology (EIT).

[20] Chordiya A.R., Majumder S., Javaid A.Y. Man-in-the-middle (mitm) attack based hijacking of http traffic using open source tools. Proceedings of the 2018 IEEE International Conference on Electro/Information Technology (EIT).

[21] Green I. (2005). DNS Spoofing by the Man in the Middle. https://www.sans.org/white-papers/1567/.

[22] Tripathi N., Swarnkar M., Hubballi N. DNS spoofing in local networks made easy. Proceedings of the 2017 IEEE International Conference on Advanced Networks and Telecommunications Systems (ANTS).

[23] Hastings N.E., McLean P.A. TCP/IP spoofing fundamentals. Proceedings of the 1996 IEEE Fifteenth Annual International Phoenix Conference on Computers and Communications.

[24] WiFi Eavesdropping: Attack Overview and Challenegs. https://www.ukessays.com/essays/computer-science/wifi-eavesdropping-attack-overview-and-challenegs.php.

[25] Acar A., Fereidooni H., Abera T., Sikder A.K., Miettinen M., Aksu H., Conti M., Sadeghi A.R., Uluagac S. Peek-a-boo: I see your smart home activities, even encrypted!. Proceedings of the 13th ACM Conference on Security and Privacy in Wireless and Mobile Networks.

[26] Zhao F., Hori Y., Sakurai K. Analysis of privacy disclosure in DNS query. Proceedings of the 2007 International Conference on Multimedia and Ubiquitous Engineering (MUE’07).

[27] Velan P., Čermák M., Čeleda P., Drašar M. (2015). A survey of methods for encrypted traffic classification and analysis. Int. J. Netw. Manag..

[28] Kotak J., Elovici Y. Iot device identification using deep learning. Proceedings of the 13th International Conference on Computational Intelligence in Security for Information Systems (CISIS 2020) 12.

[29] Tang J., Yang L., Liu S., Liu W., Wang M., Wang C., Jiang B., Lu Z. Caps-LSTM: A Novel Hierarchical Encrypted VPN Network Traffic Identification Using CapsNet and LSTM. Proceedings of the International Conference on Science of Cyber Security.

[30] Nassi B., Pirutin Y., Shamir A., Elovici Y., Zadov B. (2020). Lamphone: Real-time passive sound recovery from light bulb vibrations. Cryptol. ePrint Arch..

[31] Nassi B., Pirutin Y., Galor T., Elovici Y., Zadov B. Glowworm Attack: Optical TEMPEST Sound Recovery via a Device’s Power Indicator LED. Proceedings of the 2021 ACM SIGSAC Conference on Computer and Communications Security.

[32] Callegati F., Cerroni W., Ramilli M. (2009). Man-in-the-Middle Attack to the HTTPS Protocol. IEEE Secur. Priv..

[33] Pateriya P.K., Kumar S.S. (2012). Analysis on Man in the Middle Attack on SSL. Int. J. Comput. Appl..

[34] Mozilla Use Primary Password to Protect Stored Logins. https://support.mozilla.org/en-US/kb/use-primary-password-protect-stored-logins.

[35] Matsui M. Linear cryptanalysis method for DES cipher. Proceedings of the Workshop on the Theory and Application of of Cryptographic Techniques.

[36] Nahari S. (2021). Best Defense? Our Red Team Lead Reveals 4 MFA Bypass Techniques. https://www.cyberark.com/resources/threat-research-blog/mfa-bypass-techniques-from-red-team-research.

[37] Tassey M., Perkins R. Wireless aerial surveillance platform. Proceedings of the DEFCON Conference.

[38] Reed T., Geis J., Dietrich S. {SkyNET}: A {3G-Enabled} Mobile Attack Drone and Stealth Botmaster. Proceedings of the 5th USENIX Workshop on Offensive Technologies (WOOT 11).

[39] Ronen E., Shamir A., Weingarten A.O., O’Flynn C. IoT goes nuclear: Creating a ZigBee chain reaction. Proceedings of the 2017 IEEE Symposium on Security and Privacy (SP).

[40] Greenberg A. (2019). Watch a Drone Take over a Nearby Smart TV. https://www.wired.com/story/smart-tv-drone-hack/.

[41] Toh J., Hatib M., Porzecanski O., Elovici Y. Cyber security patrol: Detecting fake and vulnerable wifi-enabled printers. Proceedings of the Symposium on Applied Computing.

[42] Nassi B., Shabtai A., Masuoka R., Elovici Y. (2019). SoK-security and privacy in the age of drones: Threats, challenges, solution mechanisms, and scientific gaps. arXiv.

[43] Iperov Real-Time Face Swap for PC Streaming or Video Calls. https://github.com/iperov/DeepFaceLive.

[44] Alievk Avatars for Zoom, Skype and Other Video-Conferencing Apps. https://github.com/alievk/avatarify-python.

[45] CorentinJ Clone a Voice in 5 Seconds to Generate Arbitrary Speech in Real-Time. https://github.com/CorentinJ/Real-Time-Voice-Cloning.

[46] Aafer Y., You W., Sun Y., Shi Y., Zhang X., Yin H. Android {SmartTVs} Vulnerability Discovery via {Log-Guided} Fuzzing. Proceedings of the 30th USENIX Security Symposium (USENIX Security 21).

[47] Goodin D. (2017). Smart TV Hack Embeds Attack Code into Broadcast Signal—No Access Required. https://arstechnica.com/information-technology/2017/03/smart-tv-hack-embeds-attack-code-into-broadcast-signal-no-access-required/.

[48] Meier J. (2003). Improving Web Application Security: Threats and Countermeasures.

[49] Nassi B., Ben-Netanel R., Shamir A., Elovici Y. Drones’ cryptanalysis-smashing cryptography with a flicker. Proceedings of the 2019 IEEE Symposium on Security and Privacy (SP).

[50] Nuss B., Sit L., Fennel M., Mayer J., Mahler T., Zwick T. MIMO OFDM radar system for drone detection. Proceedings of the 2017 18th International Radar Symposium (IRS).

[51] England P., Malvar H.S., Horvitz E., Stokes J.W., Fournet C., Burke-Aguero R., Chamayou A., Clebsch S., Costa M., Deutscher J. Amp: Authentication of media via provenance. Proceedings of the 12th ACM Multimedia Systems Conference.

[52] Yousuf B., Qureshi M.A., Spillane B., Munnelly G., Carroll O., Runswick M., Park K., Culloty E., Conlan O., Suiter J. (2021). PROVENANCE: An Intermediary-Free Solution for Digital Content Verification. arXiv.

[53] Li Y., Lyu S. (2018). Exposing deepfake videos by detecting face warping artifacts. arXiv.

[54] Ciftci U.A., Demir I., Yin L. Fakecatcher: Detection of synthetic portrait videos using biological signals. IEEE Trans. Pattern Anal. Mach. Intell..

[55] Li L., Bao J., Zhang T., Yang H., Chen D., Wen F., Guo B. Face x-ray for more general face forgery detection. Proceedings of the IEEE/CVF Conference on Computer Vision and Pattern Recognition.

[56] Bolles R.C., Burns J.B., Graciarena M., Kathol A., Lawson A., McLaren M., Mensink T. Spotting Audio-Visual Inconsistencies (SAVI) in Manipulated Video. Proceedings of the CVPR Workshops.

[57] Güera D., Delp E.J. Deepfake video detection using recurrent neural networks. Proceedings of the 2018 15th IEEE International Conference on Advanced Video and Signal Based Surveillance (AVSS).

[58] Agarwal S., Farid H., Gu Y., He M., Nagano K., Li H. Protecting World Leaders Against Deep Fakes. Proceedings of the CVPR Workshops.

[59] Marra F., Gragnaniello D., Verdoliva L., Poggi G. Do gans leave artificial fingerprints?. Proceedings of the 2019 IEEE Conference on Multimedia Information Processing and Retrieval (MIPR).

[60] Xuan X., Peng B., Wang W., Dong J. On the generalization of GAN image forensics. Proceedings of the Chinese Conference on Biometric Recognition.

[61] Tolbert M. (2021). Vulnerabilities of Multi-factor Authentication in Modern Computer Networks. Ph.D. Thesis.

[62] Künnemann R., Steel G. YubiSecure? Formal security analysis results for the Yubikey and YubiHSM. Proceedings of the International Workshop on Security and Trust Management.

[63] Ren J., Dubois D.J., Choffnes D., Mandalari A.M., Kolcun R., Haddadi H. Information exposure from consumer iot devices: A multidimensional, network-informed measurement approach. Proceedings of the Internet Measurement Conference.

[64] Mohammadmoradi H., Gnawali O. Making whitelisting-based defense work against badusb. Proceedings of the 2nd International Conference on Smart Digital Environment.

[65] Microsoft Inture—Restrict USB. https://docs.microsoft.com/en-us/troubleshoot/mem/intune/restrict-usb-with-administrative-template.

[66] Kakarla S.K.R., Tang A., Beckett R., Jayaraman K., Millstein T., Tamir Y., Varghese G. Finding network misconfigurations by automatic template inference. Proceedings of the 17th USENIX Symposium on Networked Systems Design and Implementation (NSDI 20).

[67] Harikrishnan N., Vinayakumar R., Soman K. A machine learning approach towards phishing email detection. Proceedings of the Anti-Phishing Pilot at ACM International Workshop on Security and Privacy Analytics (IWSPA AP).

[68] Gangavarapu T., Jaidhar C., Chanduka B. (2020). Applicability of machine learning in spam and phishing email filtering: Review and approaches. Artif. Intell. Rev..

[69] Smadi S., Aslam N., Zhang L. (2018). Detection of online phishing email using dynamic evolving neural network based on reinforcement learning. Decis. Support Syst..

[70] He Z., Zhang T., Lee R.B. Machine learning based DDoS attack detection from source side in cloud. Proceedings of the 2017 IEEE 4th International Conference on Cyber Security and Cloud Computing (CSCloud).

[71] Yuan X., Li C., Li X. DeepDefense: Identifying DDoS attack via deep learning. Proceedings of the 2017 IEEE International Conference on Smart Computing (SMARTCOMP).

[72] Trusted App Protection (TAP). https://www.beyondtrust.com/docs/privilege-management/windows/admin/policies-and-templates/templates/trusted-app-protection.htm.

[73] Lee P.S. (2019). ONP: Man-in-the-Middle Attack Prevention (Early Availability). https://community.checkpoint.com/t5/Mobile/ONP-Man-in-the-Middle-attack-prevention-early-availability/m-p/41710.

[74] Mirsky Y., Kalbo N., Elovici Y., Shabtai A. (2018). Vesper: Using echo analysis to detect man-in-the-middle attacks in LANs. IEEE Trans. Inf. Forensics Secur..

[75] Gupta A.A. (2017). Length Hiding VPN to Mitigate Compression Side-Channel and Traffic Analysis Attacks. Ph.D. Thesis.

[76] Panda S. (2022). Experience a Faster and More Private Internet in Library and Information Centres with 1.1. 1.1 DNS Resolver. Int. J. Smart Sens. Adhoc Netw..

